# miRNAs and lncRNAs: Potential Non-Invasive Biomarkers for Endometriosis

**DOI:** 10.3390/biomedicines9111662

**Published:** 2021-11-11

**Authors:** Ioana Maria Maier, Adrian Cornel Maier

**Affiliations:** 1 Emergency Military Hospital, 800150 Galati, Romania; mioanamaier@gmail.com; 2 Faculty of Medicine and Pharmacy, Dunarea de Jos University, 800008 Galati, Romania

**Keywords:** miRNAs, lncRNAs, endometriosis

## Abstract

Many studies have tried to understand the mechanism of endometriosis and its manner of manifestation. However, the only method of diagnosis considered as the gold standard in endometriosis is an invasive method called exploratory laparoscopy. Hence, there is a need to identify non-invasive or minimally invasive methods to minimize patients’ suffering, thus increasing their addressability at the earliest possible staging of the disease, and to diagnose this condition as soon as possible. miRNAs (microRNAs) and lncRNAs (long-noncoding RNAs) are potential non-invasive diagnostic methods for endometriosis. Multiple clinical trials indicate that miRNA can be used as a non-invasive method in the diagnosis and differentiation of endometriosis stages.

## 1. Introduction

Endometriosis is a gynecologic disease characterized by the presence of glandular epithelium and endometrium-like stroma outside of the uterine cavity [1,2]. It is a benign estrogen-dependent inflammatory disorder. Lesions can be identified in the peritoneum and ovary (endometrioma or superficial implants), and deep lesions can extend to the bladder, ureter, and bowel [3,4,5].

The most frequent symptoms and signs are as follows: dysmenorrhea, pelvic pain, dysuria, and, in many cases, infertility [6,7,8].

Due to the misinterpretation of endometriosis-induced pain, the diagnosis of endometriosis is delayed for 8–10 years [9]. Laparoscopic surgery remains the gold standard for the diagnosis of endometriosis, with histopathological confirmation in the excised lesions [10,11].

Endometriosis has been classified as a tumor-like condition in the WHO histological classification of ovarian tumors [12]. It is known that a lot of common features are present in endometriosis and ovarian carcinoma, including the following: increased local estrogen production, inflammation, tissue invasion, disfunction of immune cells, and induction of angiogenesis [13].

Many theories have been proposed for the pathogenesis of endometriosis, such as retrograde menstruation, celomic metaplasia, and lymphatic and hematic invasion, but the most accepted theory is Sampson’s retrograde menstruation [14].

Non-coding RNAs are RNA fragments that are transcribed from DNA, but are not translated into proteins. Non-coding RNAs can be divided into the following two groups according to their length: short non-coding RNAs (<30 nucleotides); long non-coding RNAs (>200 nucleotides) [15]. Recent studies showed that miRNA and lncRNA can be used in the diagnosis, treatment, and prognosis of gynecological cancer and endometriosis [16,17,18,19].

Currently, the research of biomarkers in endometriosis is still lacking reproducible data with high sensitivity and specificity. Achieving high specificity and sensitivity of non-invasive biomarkers is a challenge for endometriosis because of the heterogeneity of the disease characteristics, and because of the comorbidities suffered by endometriosis patients [20,21,22].

According to Anastasiu et al., a panel of miRNAs or lncRNAs could be more conducive indicators of this gynecological pathology [23].

Several studies found miRNA to be representative in various samples, such as urine, serum, plasma, and other fluids of the human body, so it can be considered as a biomarker [24,25,26]. The detection of a small number of miRNAs gives more information about the disease than studying the expression of several mRNAs [27]. The potential of using miRNAs as biomarkers for endometriosis detection is also more attractive due to their lower complexity and lack of known post-translational modifications [28]. Despite the limitations, such as a variable GC (guanine–cytosine) content, miRNAs have opened new horizons and have given a new dimension to the field of biomarkers [29].

## 2. Biogenesis of miRNAs

MicroRNAs (miRNAs) are small, single-stranded, non-coding RNA molecules (18–25 nucleotides) that ensure the post-transcriptional regulation of gene expression by the inhibition of translation and/or the promotion of messenger RNA degradation (mRNA), due to partial or complete pairing with 3′ UTR regions of mRNA [30,31]. Together with other molecular mechanisms, miRNAs are potent post-transcriptional gene regulators [32]. In the nucleus, miRNAs are transcribed by RNA polymerase II as pri-miRNA [33], and further processed into pre-miRNA by the microprocessor complex, composed of RNase III Drosha together with its DGCR8 regulatory subunit [34,35]. Pre-miRNAs are then actively transported into the cytoplasm by Exportin-5 [36], where they are cleaved into miRNA duplex intermediates by RNase III Dicer. Then, the conducting miRNA strand is selected and loaded into the miRNA-induced silencing complex (miRISC) that regulates the expression of target genes [37]. miRNAs can be exported to the extracellular space, and can circulate in the bloodstream associated with microparticles, exosomes, lipoproteins, or protein complexes, which can act as long-distance extracellular messengers [38,39,40,41]. MicroRNAs plays important roles in pathology, being stress response molecules and having modified expressions during the evolution of diseases [42,43]. The modified cellular miRNA expression and altered circulating miRNA profile have been associated with various disorders, including different cancers, endometriosis, neuro-degenerative diseases, atherosclerosis, obesity, diabetes, or coronary heart disease [44,45,46,47,48]. The circulating miRNA profile may reflect altered tissue expression or intercellular communication, suggesting their possible use as biomarkers [49].

It is important to highlight that the DNA sequence is not always the template for mature miRNA. Approximately 6% of human miRNAs suffer from RNA editing, so a single pre-miRNA becomes multiple mature miRNAs that are different in length and sequence, and are given the name isomiR [15,50]. The cell-specific expression of different isomiRs implies another protein expression depending on the cell type, with a high spectrum of miRNA action [51].

The first aim in a study is to evaluate the miRNA expression by comparing it to a control group and a pathological group. The techniques that assess the miRNA expression profiles are as follows: RNA sequencing, miRNA microarrays, qPCR (quantitative PCR) or RT-PCR (real-time PCR), ISU (in situ hybridization), and live-cell miRNA detection. RNA sequencing was developed for sequencing genes faster, deeper, and with a lower cost. The sensibility of this technique is linked to coverage and throughput, so it is possible that overexpressed miRNAs reduce the ability to detect miRNAs that are very lowly expressed [51].

Microarray technology is based on the analysis of millions of genomic fragments on a glass support used for sample hybridization. The samples are fluorescently labeled before hybridization, so that a signal in each probe reflects the number of miRNAs from the samples. This technique must be confirmed by qPCR. The qPCR method consists of the synthesis of complementary DNA to miRNA in the presence of a retrotranscription protocol. The process in RT-PCR is sensitive and a low level of expression can be detected, which means that it is the best method for the detection and quantification of miRNAs in, for example, plasma and serum. So far, qPCR represents one of the best reference methods for expression and validation in miRNA [52].

## 3. miRNAs Expression in Endometrium Samples

Many studies have been focused on the potential role of miRNAs in endometriosis. The characteristics of miRNAs give them the ability to act as a marker in terms of the presence of endometriosis, or even establish the stage of the disease. miRNAs are present in various liquid substances produced in the human body, such as plasma, serum, and peritoneal fluid. They are also found in fresh frozen tissues, or in formalin-fixed paraffin-embedded tissues. The structural integrity of miRNA is due to the increased stability of RNA chains under different conditions [53].

The altered miRNA expression in patients with endometriosis may be considered as a promoter for the development of ovarian carcinoma [54].

Burney et al. published the first study on miRNA expression in the endometrium of women with and without endometriosis. In this study, the authors reported a reduction in the levels of four miRNAs (miR-34c-5p, miR-9, miR-9* and miR-34b*) in the endometrium, from the patients with endometriosis compared to the control group. It is considered that the overexpression of miRNAs in endometriosis may be responsible for stimulating the development of endometriosis [38]. Previous studies have shown the expression of miRNAs in endometrial implants and in the eutopic endometrium of women with and without associated endometriosis [52,55].

Filigheddu et al. (2010) investigated the different expressions of miRNAs in endometriosis by comparing ectopic and endometrial tissue samples from the endometrium. Thus, about 50 miRNAs were found to be differently expressed, and in the case of five miRNAs (miR-200a, miR-200b, miR-200c, miR-182, and miR-202), these were confirmed by RT-PCR in 13 patients. The levels of miR-200a, miR-200b, miR-200c, and miR-182 were reduced to 95%, and miR-202 in the ectopic endometrium was 60 times higher than in the eutopic endometrium [56].

The secretory eutopic endometrium of patients with endometriosis is characterized by different miRNA expression to that of the secretory endometrium without endometriosis present in these patients. The members of the miR-9 and miR-34 families are poorly regulated in the endometrium of patients with endometriosis compared to those without this condition. Hawkins et al. reported 10 miRNAs that were more expressed (miR-202, miR-193a-3p, miR-29c, miR-708, miR-509-3-5p, miR-574-3p, miR-193a-5p, miR-485-3p, miR-100, and miR-720) and 12 miRNAs with reduced or poorly expressed levels (miR-504, miR-141, miR-429, miR-203, miR-10a, miR-200b, miR-873, miR-200c, miR-200a, miR-449b, miR-375 and miR-34c-5p) in ovarian endometrial cysts compared to the endometrium [52].

In addition, Ohlsson and colleagues conducted a comparative study on the expression of miRNAs in the eutopic and ectopic endometrium, and found the following: 14 intensely regulated miRNAs (miR-145, miR-143, miR-99a, miR-99b, miR-126, miR-100, miR-125b, miR-223, miR-194, miR-365, miR-29c, and miR-1), and 8 poorly regulated miRNAs (miR-200a, miR-141, miR-200b, miR-142-3p, miR-424, miR-34c, miR-20a, and miR-196b). The miR-200 family (miR-200a, miR-200b, and miR-141) was intensively studied, and the same author identified a sensitivity of 84.4% and a specificity of 66.7% in patients with endometriosis (Table 1) [57].

In another study, which included 25 patients without endometriosis and 21 patients with endometriosis, the expression of 667 miRNAs was studied by the PCR method. miR-483-5p and miR-629-3p were poorly regulated in the eutopic endometrium compared to the control group, so it was concluded that disorders of these genes may contribute to the growth of endometrial tissue outside the uterus [58].

## 4. The Role of Circulating miRNAs in Endometriosis

The use of circulating miRNAs as biomarkers of endometriosis is an important field of research; however, only few data are known and have been published on the presence of miRNAs in serum and plasma [58,59,60].

The first study about circulating miRNAs was performed by Wang et al., with the following two lots: one containing 10 patients with endometriosis, and another with 10 control patients. A total of 765 miRNAs were identified using a TaqMan miRNA array in a pool, and a set of selected miRNAs were further analyzed in a validation cohort consisting of sera from 60 patients and 25 controls, including 10 samples used in array profiling. The miR-199a and miR-122 levels were upregulated, and miR-145*, miR-141*, miR-542-3p, and miR-9* were downregulated compared with the control group. The same study reported that a panel of four miRNAs (miR-122, miR-145, miR-199a, miR-542-3p) had 93.2% specificity and 96% sensitivity in detecting women with endometriosis [61].

Another study, conducted by Jia et al., included 23 women with endometriosis that was initially confirmed by laparoscopy and then, finally, by histopathology exam, and 23 without endometriosis. Twenty-seven microRNAs were differentially expressed between women with and without endometriosis, of which six microRNAs (miR-15b-5p, miR-17-5p, miR-20a, miR-21, miR-22, and miR-26a) were selected for validation. Three miRNAs (miR-17-5p, miR-20a, and miR-22) were significantly downregulated in patients with endometriosis compared with controls (Table 2) [59].

In 2015, Cho et al. published a study that included 24 women with endometriosis and 24 women without the disease (control group). Serum samples were collected from women undergoing laparoscopy for endometriosis and other benign gynecological diseases. The results showed that the levels of circulating let-7b and miR-135a were statistically significantly lower in women with endometriosis compared with controls. The expression of let-7b was strongly correlated with serum CA-125 levels. When the patients were analyzed according to the phase of their menstrual cycle, the expressions of let-7b, let-7c, let-7d, and let-7e were statistically significantly lower in the women with endometriosis during the proliferative phase. The sensitivity and specificity of let-7b were 83.3% and 100%, respectively, in the diagnosis of endometriosis [58].

In the same year, Rekker et al. published a study that included 61 patients with endometriosis, confirmed by laparoscopic examination, patients without endometriosis, and 30 healthy women. The results showed that the levels of the miRNAs that were studied varied with blood collection time, being lower in the morning than in the evening; the values revealed significantly lower levels of miR-200a and miR-141 in the evening plasma samples of the women with endometriosis compared with the patients without endometriosis. The evening sample levels of miRNAs were lower in the patients with stage I–II endometriosis than in the control subjects. In the patients with stage III–IV endometriosis, only miR-200a was significantly lower compared with the patients without endometriosis [60].

Kozomara et al. compared the values of miR-145 between the women with endometriosis and the healthy women, and found that miR-145 is upregulated in the advanced stages of endometriosis and downregulated in the early stages [62]. In their study, Bashti et al. found that miR-31 and miR-145 are involved in endometriosis; they used plasma samples from 55 patients with endometriosis, 34 patients with histologically proven stage 3 or 4 endometriosis (moderate or severe forms), 21 patients with stage 1 or 2 endometriosis (minimal and mild forms), and 23 patients without endometriosis. The expression levels of miR-31 in stage 3 or 4, and stage 1 or 2, were downregulated, and the level of miR-145 was significantly upregulated in patients with stage 1 or 2 endometriosis [63].

In another study, Nisenblat et al. identified forty-nine miRNAs that were differentially expressed in patients with endometriosis. Three of these miRNAs (miR-155, miR-574-3p, and miR-139-3p) demonstrated a sensitivity and specificity of 83% and 51%, respectively [64].

In the same year as Nisenblat (2019), Vanhie et al. studied 42 miRNAs, and only three miRNAs (hsa-miR-125b-5p, hsa-miR-28-5p, and hsa-miR-29a-3p) from the total were confirmed, by RT-qPCR, to have a diagnostic power above chance performance in the independent validation, with an acceptable sensitivity (78%), but poor specificity (37%) [65].

In 2018, Maged et al. published a study evaluating the value of miR-122 and miR-199a in 45 patients with endometriosis and 35 patients with pelvic pain, confirmed by laparoscopy examination. Serum miR-122 and miR-199a had a sensitivity of 95.6% and 100%, and a specificity of 91.4% and 100%, respectively, for the diagnosis of endometriosis [66]. Moustafa et al. have shown,, in 100 patients (mean age of 34.1–36.9) with endometriosis, significantly higher expression levels of the following four serum microRNA samples: miR-125b-5p, miR-150-5p, miR-342-3p, and miR-451a. The following two serum microRNA samples showed lower levels in the endometriosis group: miR-3613-5p and let-7b. Apparently, this is the first report showing that microRNA biomarkers can differentiate between endometriosis and other gynecological pathologies [67].

In 2018, in a prospective cohort study, Pateisky et al. found that specific plasma miRNA characteristics were associated with endometriosis, and hsa-miR-154-5p alone, or with another type of miRNA, can be potentially applicable for the non-invasive diagnosis of the disease [68].

In various studies, Agrawal et al. found a total of 40 miRNAs that were differentially regulated in the circulation of women with endometriosis [27]. Hudson showed that a specific plasma miRNA signature is associated with endometriosis, with miR-154-5p alone, or in combination with miR-196b, miR-378-3p, and miR-33a-5p. With this study, the total number of studies that evaluate the potential of circulating miRNAs as a diagnosis for endometriosis is 15 [69].

## 5. lncRNAs and Endometriosis

One new potential biomarker is lncRNA (long non-coding RNA); lncRNAs are a class of molecules with a length of more than 200 nucleotides. Similarly to miRNAs, lncRNAs are studied for their involvement in many biological processes [70]. Among non-coding RNAs, lncRNAs are the most common. In total, 8801 small non-coding RNAs (<30 nt) and 9640 long non-coding RNAs (>200 nt) are known to be present in the human genome [71].

Long non-coding RNAs represent the highest proportion of ncRNAs. Most lncRNAs have the ability to regulate DNA replication, RNA transcription, and protein translation through complementary pairing with microRNAs.

Based on their location in the genome, lncRNAs can be classified into the following types: (a) long intergenic non-coding RNAs (lincRNAs); (b) natural antisense transcripts (NATs); (c) intronic lncRNAs, transcribed from intergenic regions of the genome by RNA polymerase II [72]. lncRNAs play an important role in pathogenesis and the development of diseases [73].

H19 is a 2–3 kb lncRNa located in human chromosome 11p15.5, which can be transcribed, but not translated, and H19 and insulin-like growth factor 2 (IGF2) form a pair of imprinted genes [7]. The first research of lncRNA at the level of the eutopic endometrium showed a reduction in H19 lncRNA levels in the case of patients with endometriosis. The decreased H19 led to increased let-7 activity, which inhibits IGF1 R (insulin-like growth factor 1 receptor). This process reduces the proliferation of endometrial stromal cells. These results represent the first example of the implications of lncRNA in endometriosis and its associated infertility, and inform the future development of novel therapeutics for women with endometriosis and infertility [74].

In the same year (2015), Wang et al. published a study where they compared the expression of lncRNAs and mRNAs between the eutopic and normal endometrium (both are late secretory) by microarray analysis and RT-qPCR validation. They found eight lncRNAs and mRNAs differentially expressed between the eutopic and normal endometrium. The 1277 dysregulated lncRNAs (488 upregulated and 789 downregulated) and 1216 mRNAs (578 upregulated and 638 downregulated) were expressed differentially. AC068282.3 is upregulated and RP11-403H13.1 is most significantly downregulated [75].

MALAT1 is another important lncRNA in endometriosis that cannot be increased. Some authors showed that miR-200c, which is regulated by MALAT1, was downregulated in the endometrial tissue of patients with endometriosis. miR-200c is a class of small, non-coding, single-strapped RNAs that are 20–24 nucleotides in length [76]. By using a rat endometriosis model, it is shown that the local delivery of the miR-200c mimic cannot inhibit the growth of endometriosis [77].

Li et al. supposed that lncRNA MALAT-1 is located in the nucleus of granulosa cells with endometriosis, while the expression of lncRNA MALAT1 was negatively correlated with the expression of P21 [78].

A large number of studies confirmed that the gene is related to the recurrences, metastasis, and epithelial–mesenchymal transformation of various tumors [79,80].

Wang et al. (2016) found that a unique set of lncRNAs in serum were associated with disease severity and progression; these lncRNAs were found to discriminate between the early stages and severe stages, and their diagnostic values were also investigated. This study demonstrated that lncRNAs could be non-invasive biomarkers for the diagnosis of endometriosis, and act as important regulators in the progression of this disease. Five lncRNAs can discriminate between the patients with and without endometriosis; lncRNA ENST00000482343 presents the highest sensitivity (72.41%) and specificity (71.74%) (Table 3) [70].

Huang et al. (2019) found that the downregulation of lncRNA UCA1 cannot be a biomarker for the diagnosis of ovarian endometriosis. They indicated that, for most patients with endometriosis, the expression of UCA1 was upregulated in ectopic tissue, in comparison with that in eutopic endometrial tissue. Most importantly, on the day of discharge, the serum levels of UCA1 were downregulated in patients with recurrence, in comparation with patients without recurrence [19]. More recently, Qiu et al. (2020) indicated lncRNA TC0101441 as a potential extracellular vesicular biomarker for endometriosis. It was shown that extracellular vesicular TC0101441 serum levels increased substantially in patients at stage 3/4 endometriosis in comparison with patients at stage 1/2 endometriosis and the controls; this highlights the potential of circulating extracellular vesicular TC0101441 as a biomarker for endometriosis [81].

## 6. Conclusions

Little is known about the etiopathogenesis of endometriosis. Although the prevalence of the disease is relatively high, affecting approximately 10% of sexually active females, there may be a delay in diagnosis of many years (8–10 years). Biomolecular markers are a starting point for an earlier diagnosis. Knowing that laparoscopy examination is the gold standard in the diagnosis of endometriosis, which is an invasive method, we consider that it is necessary to identify non-invasive or minimally invasive methods in the early diagnosis of endometriosis.

However, the sensitivity and specificity of these studies are not so high. The diagnosis of endometriosis may not only be based on these analyses, but a determination of both miRNAs and lncRNAs is likely to lead to an increase in their values of sensitivity and specificity. Exploratory laparoscopy may be replaced, in the future, by these investigations, as a first step in diagnosing the disease. The studies of Suryawanshi, Jia, Wang, Rekker, and Cosar revealed that some groups of dysregulated miRNAs have higher sensibility and specificity, and can represent a starting point for highlighting endometriosis, differentiation between the stages of this disease, and the identification of recurrences. The fact that the determination of these biomarkers is made by non-invasive methods makes the addressability of women much higher, leading to an earlier diagnosis, a faster intervention, and an increase in the quality of life for these women. This review attempts to present the important role that miRNAs and lncRNAs can play in diagnosing endometriosis, and also their role in differentiating the different stages in the evolution of this disease, with both being non-invasive methods that can be evaluated both together and separately.

## Figures and Tables

**Table 1:** Downregulation and upregulation of miRNAs expressed in different samples.

miRNA	Downregulation	Sensitivity (%)	Specificity (%)	miRNA	Upregulation	Sensitivity (%)	Specificity (%)
miR-9	Down	N/A	N/A	miR-29c	up	N/A	N/A
miR-9*	Down	N/A	N/A	miR-365	up	N/A	N/A
miR-20a	Down	60	90	miR-194	up	N/A	N/A
miR-34b	Down	N/A	N/A	miR-223	up	N/A	N/A
miR-34c-5p	Down	N/A	N/A	miR-125-b	up	56.1	78
miR-34b*	Down	N/A	N/A	miR-100	up	N/A	N/A
miR-23b	Down	N/A	N/A	miR-126	up	N/A	N/A
miR-17-5p	Down	60	90	miR-99a	up	N/A	N/A
miR-141	Down	71.9	70.8	miR-99b	up	N/A	N/A
miR-142-3p	Down	N/A	N/A	miR-143	up	N/A	N/A
miR-182	Down	N/A	N/A	miR-145	up	70	96
miR-196b	Down	N/A	N/A	miR-720	up	N/A	N/A
miR-200a	Down	90.6	62.5	miR-485-3p	up	N/A	N/A
miR-200b	Down	90.6	70.8	miR-202	up	N/A	N/A
miR-200c	Down	N/A	N/A	miR-193a-3p	up	N/A	N/A
miR-424	Down	N/A	N/A	miR-29c	up	N/A	N/A
miR-483-5p	Down	N/A	N/A	miR-708	up	N/A	N/A
miR-542-3p	Down	79.66	92	miR-509-3-5p	up	N/A	N/A
miR-449b	Down	N/A	N/A	miR-574-3p	up	N/A	N/A
miR-375	Down	N/A	N/A	miR-193a-5p	up	N/A	N/A
miR-10a	Down	N/A	N/A				
miR-429	Down	N/A	N/A				
miR-203	Down	N/A	N/A				
miR-504	Down	N/A	N/A				
miR-873	Down	N/A	N/A				
miR-629-3p	Down	N/A	N/A				

Legend: N/A—not available data; miR-9*—represent miR-9 family; miR-34b*—represent miR-34 family.

**Table 2:** miRNAs differentially expressed in serum samples of patients with endometriosis.

miRNA	Expression	Sensitivity (%)	Specificity (%)
let-7b	down	82.5	67.8
let-7d	down	83.3	100
let-7f	down	N/A	N/A
miR-135a	down	N/A	N/A
miR-141*	down	71.69	96
miR-145*	down	70	96
miR-542-3p	down	79.66	92
miR-9*	down	N/A	N/A
miR-17-5p	down	N/A	N/A
miR-20a	down	N/A	N/A
miR-22	down	N/A	N/A
miR-145	down (early stages)	N/A	N/A
miR-122	up	N/A	N/A
miR-145	up (advance stages)	N/A	N/A
miR-199a	up	78.33	76

Legend: miR-141*—represent miR-141 family; miR-145*—represent miR-145 family; miR-9*—represent miR-9 family.

**Table 3:** lncRNAs expressed in different samples on patients with endometriosis.

LncRNAs	Expression	Sample
AC068282.3	up	endometrium
ENST00000482343	down	serum
H19	down	endometrium
MALAT1	up	endometrium
RP11-403H13.1	down	endometrium

## Data Availability

Not applicable.
